# The relationship between anogenital HPV types and incident HIV infection among men who have sex with men and transgender women in Lima, Peru: Findings from a prospective cohort study

**DOI:** 10.1371/journal.pone.0204996

**Published:** 2018-10-02

**Authors:** Brandon Brown, Logan Marg, Segundo Leon, Cynthia Chen, Junice Ng Yi Siu, Gino Calvo, Hugo Sánchez, Jerome T. Galea

**Affiliations:** 1 School of Medicine, University of California, Riverside, Riverside, California, United States of America; 2 Socios En Salud, Lima, Peru; 3 Saw Swee Hock School of Public Health, National University of Singapore, Singapore; 4 Epicentro Salud, Lima, Peru; 5 School of Social Work, College of Behavioral and Community Sciences, University of South Florida, Tampa, Florida, United States of America; University of New South Wales, AUSTRALIA

## Abstract

Although it is known that individuals living with HIV have a higher HPV prevalence, the impact of individual HPV types on HIV acquisition is less clear. In this prospective cohort study we investigated the relationship between HPV types and incident HIV infection among men who have sex with men (MSM) and transgender women (TW) in Lima, Peru. Six hundred HIV-negative Peruvian MSM and TW participated in a 2-year study with biannual visits. At baseline, participants completed a computerized, self-administered questionnaire on sexual behavior and HPV knowledge and underwent a physical exam including anogenital swabs for HPV DNA (37 genotypes via linear array testing) and HIV testing; follow-up visits included the questionnaire and HIV testing. Participant mean age was 25 years (range = 18–40), with 48.9% self-identifying as gay and 86.5% reporting having sex exclusively with men. At baseline, 530 participants had HPV DNA present (61.1% with high-risk HPV, 84.9% with low-risk HPV). Among 571 participants who returned for any study visit, 73 (12.8%) became infected with HIV during the 2-year follow-up (6% HIV incidence). Compared to those without HIV, more participants with HIV had any HPV type present (97.3% vs. 87.6%, respectively, *p* = .01), more than one HPV type (79.5% vs. 58.2%, *p* < .01), or high-risk HPV (72.6% vs. 51.4%, *p* < .01). Some participants lost to follow-up could have been HIV-positive, which would have affected the relationship of HPV and HIV infection. Our prospective study showed that participants with any HPV type, more than one HPV type, or high-risk HPV were more likely to test positive for HIV. Although most studies have shown HPV–HIV coinfection, our findings illustrate the strong relationship between individual HPV types and HIV infection. This further illustrates the potential utility of HPV vaccine for MSM and TW, not only for HPV prevention but also possibly for HIV prevention.

## Introduction

The association of many sexually transmitted infections (STIs) with HIV acquisition among men who have sex with men (MSM) and transgender women (TW) is well established [1–3]. Testing and treatment of ulcerative STIs, in particular, is a core element of HIV prevention programs in these populations [4]. Human papillomavirus (HPV) is the most prevalent STI worldwide among all sexually active adults and affects approximately 50% of HIV-negative MSM, which may include TW because the groups are often erroneously combined [5]. Although HPV is independently associated with HIV infection in MSM [6,7] (data unavailable for TW), little is known about the relationship between specific HPV types and incident HIV infection [6].

Most new HIV infections in Latin America occur among MSM and TW, with transmission mainly occurring during unprotected anal intercourse [1,8]. In Peru, the HIV prevalence for MSM is 9.5%–10.5% [9,10], whereas the prevalence for TW is even higher, at 30% [11], far exceeding the 0.3% infection rate found in the general population (adults aged 15–49 years) [12].

Several studies have shown that high-risk HPV (HRHPV) infection and prevalence of multiple HPV types is higher among HIV-positive MSM compared to HIV-negative MSM [13,14]. Estimates of HPV prevalence in the anal canal are particularly high among MSM in Peru, ranging from 77% to 97% [14,15].

One study in Peru showed HIV-positive MSM were coinfected with twice as many HPV types as their HIV-negative counterparts [15]. Because some chronic infections with HPV can be prevented with HPV vaccination, it is possible that HPV vaccination could also be promoted as part of additive HIV prevention interventions.

Although several studies have investigated HIV and HPV coinfection, a growing body of research suggests that HPV infection is implicated in the acquisition of HIV [6,7,16]. This study explored the relationship between HPV infection and HIV acquisition by specifying the HPV types associated with incident HIV infection among all participants in a prospective cohort of MSM and TW in Lima, Peru.

## Methods

MSM and TW were recruited for a cohort study examining the relationship between anogenital warts and HIV acquisition [17,18]. The protocol for this study is described in detail elsewhere [19]. Briefly, the study site was Epicentro Salud, a local community-based health center for MSM and TW. Study participants were recruited at Epicentro Salud, bars, clubs, and volleyball courts, via social media, and by snowball sampling. Inclusion criteria were: born anatomically male, aged 18–40 years, HIV-negative, had anal intercourse with a male in the past 12 months, resident of metropolitan Lima, and willing to provide blood samples and anal swabs. Participants were excluded if they were HIV positive, participated in an HIV or HPV clinical trial, had a known immunodeficiency disorder, or if they self-reported use of pre-exposure prophylaxis (PrEP). Participants were not excluded by AGW status.

Participants completed a computerized, self-administered questionnaire in which they were asked whether they experienced any symptoms of anal bleeding, anal pain or burning, anal ulcers, or sores during the prior 6 months. Next, sociobehavioral questions were asked, including usual sexual role when having sex with a man, age at first anal intercourse, and number of instances of anal intercourse in the prior 6 months. We assessed condom use during the prior 6 months and during participants’ most recent anal sex with a man. Following the questionnaire, participants underwent a physical exam to check for signs of anogenital warts. If the presence of anogenital warts was unclear, biopsies were performed to confirm whether they were present.

Participants were followed at 6, 12, 18, and 24 months post-enrollment for HIV testing and self-administered sociobehavioral interviews. HIV status was assessed using the Determine HIV-1/2 Combo Ag/Ab test and confirmed by indirect immunofluorescence assay (in-house testing at Peruvian National Institute of Health) at each visit. Referrals for care and treatment were made as necessary.

### Laboratory assays for HPV

Following previously established protocols, anogenital specimens were collected at the baseline visit using prewetted Dacron swabs from the coronal sulcus or glans penis, penile shaft, anus, and scrotum, combined into one sample per participant, and stored at -80°C [20]. DNA was extracted at the Moffitt Cancer Center (in Tampa, FL) using the QIAamp Media MDx Kit, followed by polymerase chain reaction and HPV genotyping. Samples positive for β-globin or at least one HPV genotype were considered adequate and included in the analysis (overall β-globin positivity = 98%). The Roche Linear Array assay was used to detect 37 HPV genotypes classified as high risk (oncogenic; HPV 16, 18, 31, 33, 35, 39, 45, 51, 52, 56, 58, 59, and 68) or low risk (nononcogenic; HPV 6, 11, 26, 40, 42, 53, 54, 55, 61, 62, 64, 66, 67, 69, 70, 71, 72, 73, 81, 82, 82 subtype IS39, 83, 84, and 89 [formerly CP6108]) [21]. The genotypes were further classified into HPV9 vaccine genotypes (i.e., 6, 11, 16, 18, 31, 33, 45, 52, and 58).

### Statistical analyses

HIV status and dichotomized HRHPV genotypes, HPV9 vaccine genotypes, and HP4 vaccine genotypes were examined for explanatory relationships between HPV and HIV. In bivariate analysis, categorical variables were assessed using chi-square or trend tests (Mantel-Haenszel chi-square test), whereas continuous variables were assessed using Wilcoxon rank-sum tests. Logistic regression was used to construct multivariate analysis models. Each theoretically plausible independent variable significant at *p* < .10 in the bivariate analysis was entered into the model using backward selection. These included experiencing anal bleeding during the prior 6 months, experiencing anal ulcers during the prior 6 months, usual sexual role, and sexual identity. The first variable accounting for maximum variation in the model was selected, and subsequent variables were added until there was no significant variation in the prediction of the outcome variable to obtain the most parsimonious model. Missing data were excluded from our primary analysis. We performed a sensitivity analysis by replacing missing data by the mean or highest occurrence, and controlled for missing dummy variables in the regression model. The results were consistent with our primary analysis. Models were adjusted for demographic variables (i.e., age and education level). Statistical significance was set at *p* < .05 and adjusted odds ratios (AORs) were reported using Stata 14.0 software. In the original sample size calculation of 600 participants, a sample size of 264 MSM in each group provided 80% power to detect an 8% difference in HIV incidence (15% among those with AGW, and 7% among those without AGW; Hazard Ratio: 2.1; with a type I error of 0.05). We expected 13% loss to follow-up; therefore, 300 subjects in each group were needed.

All participants provided written informed consent to participate in this study, which took place between February 15, 2012 and September 27, 2015. This study was approved by institutional review boards at the University of California, Los Angeles in the United States and Impacta Salud y Educación in Lima, Peru.

## Results and discussion

Of 756 individuals screened for potential participation, 156 were excluded due to HIV infection at baseline. Among the 600 initially HIV-negative participants, 571 completed the final visit at month 24 and had an HIV test result (Table 1). The denominators for each variable are different due to missing data. A total of 453 reported the sex of their partners in the past 6 months, of whom 87% (394) reported having sex exclusively with men and 48.9% (277) self-identified as homosexual. Of the 566 participants who self-reported their sexual identity, 472 (83.4%) were MSM (heterosexual, homosexual, bisexual); the remainder were TW. Visible anogenital warts were noted for 233 participants at baseline (60% anal only). Participant mean age was 25 years (range = 18–40). At baseline, 530 participants had any HPV DNA present (61.1% of those with HRHPV, 84.9% with low-risk HPV). The presence of any HPV4 vaccine genotype was 42.0% (21.8 with HPV6, 11.5% with HPV11, 12.8% with HPV16, and 6.75% with HPV18). Coinfection with HPV4 low-risk genotypes (presence of both 6 and 11) was found in 14 participants, whereas coinfection with HPV4 high-risk genotypes (presence of both 16 and 18) was found in six participants. Zero participants had all HPV4 genotypes present.

**Table 1:** Comparison of characteristics among Peruvian MSM and TW with and without HIV at month 24 (*N* = 571).

Characteristics	Total n (%)	HIV-Positive n (%)	HIV-Negative n (%)	*p*
Overall	571 (100)	73 (12.8)	498 (87.2)	
Age				.039
18–24	225 (46.9)	34 (51.5)	191 (46.1)	
25–29	141 (29.4)	24 (36.4)	117 (28.3)	
30+	114 (23.8)	8 (12.1)	106 (25.6)	
Education				.871
Secondary or less	271 (47.5)	34 (46.6)	237 (47.6)	
Tertiary or university	300 (52.5)	39 (53.4)	261 (52.4)	
Current STI symptoms				.038
Yes	122 (31.3)	22 (44.0)	100 (29.4)	
No	268 (68.7)	28 (56.0)	240 (70.6)	
Anal bleeding, last 6 months				.005
Yes	54 (12.9)	12 (27.9)	42 (11.2)	
No	364 (87.1)	31 (72.1)	333 (88.8)	
Anal ulcers or sores, last 6 months				.016
Yes	22 (5.1)	7 (12.3)	15 (4)	
No	411 (94.9)	50 (87.7)	361 (96)	
Current anogenital warts				.224
Yes	206 (30.1)	31 (42.5)	175 (35.1)	
No	365 (63.9)	42 (57.5)	323 (64.9)	
Sexual identity				.127
Heterosexual	54 (9.5)	7 (9.7)	47 (9.5)	
Homosexual	277 (48.9)	39 (54.2)	238 (48.2)	
Bisexual	141 (24.9)	21 (29.2)	120 (24.3)	
Transgender^a^	94 (16.6)	5 (6.9)	89 (18)	
Age at first anal intercourse				.272
< 14	105 (18.4)	10 (13.7)	95 (19.1)	
14–19	340 (59.5)	50 (68.5)	290 (58.2)	
> 19	126 (22.1)	13 (17.8)	113 (22.7)	
Instances of anal sex, last 6 months^b^	8 (4–20)	10 (3–20)	7 (4–20)	.599
Condom used, last anal sex with a man				.040
Yes	295 (72.7)	35 (61.4)	260 (74.5)	
No	111 (27.3)	22 (38.6)	89 (25.5)	
Sex of partners, last 6 months				.349
Men only	394 (87)	58 (90.6)	336 (86.4)	
Men and women only	59 (13)	6 (9.4)	53 (13.6)	
Sexual role				.040
Insertive only or mostly	112 (24.6)	9 (14.3)	103 (26.3)	
Receptive only or mostly or equally insertive and receptive	343 (75.4)	54 (85.7)	289 (73.7)	
Sex and alcohol, past month				.654
Yes	145 (30.5)	18 (28.1)	127 (30.9)	
No	330 (69.5)	46 (71.9)	284 (69.1)	
Sex and drugs, past month				.876
Yes	26 (5.6)	4 (6.3)	22 (5.5)	
No	439 (94.4)	60 (93.7)	379 (94.5)	
Transactional sex				.486
Yes	14 (28.0)	1 (50.0)	13 (27.1)	
No	36 (72.0)	1 (50.0)	35 (72.9)	
Any HPV Present				.014
Yes	507 (88.8)	71 (97.3)	436 (87.6)	
No	64 (11.2)	2 (2.7)	62 (12.5)	
At least one HRHPV				.001
Yes	309 (54.1)	53 (72.6)	256 (51.4)	
No	262 (45.9)	20 (27.4)	242 (48.6)	
At least one low-risk HPV				.418
Yes	432 (75.7)	58 (79.5)	374 (75.1)	
No	139 (24.3)	15 (20.5)	124 (24.9)	
Circumcised				.505
Yes	24 (4.2)	2 (2.7)	22 (4.4)	
No	547 (95.8)	71 (97.3)	476 (95.6)	
HPV DNA 16, 18^c^				.027
Yes	105 (18.6)	20 (28.2)	85 (17.2)	
No	459 (81.4)	51 (71.8)	408 (82.8)	
HPV DNA 16, 18, 31, 33, 35, 52, 58^c^				.021
Yes	201 (35.6)	34 (47.9)	167 (33.9)	
No	363 (64.4)	37 (52.1)	326 (66.1)	

^a^At the time of the survey we included transgender as a sexual identity.

^b^Figures reflect median (interquartile range).

^c^Presence of any of the listed genotypes.

**Fig 1:**
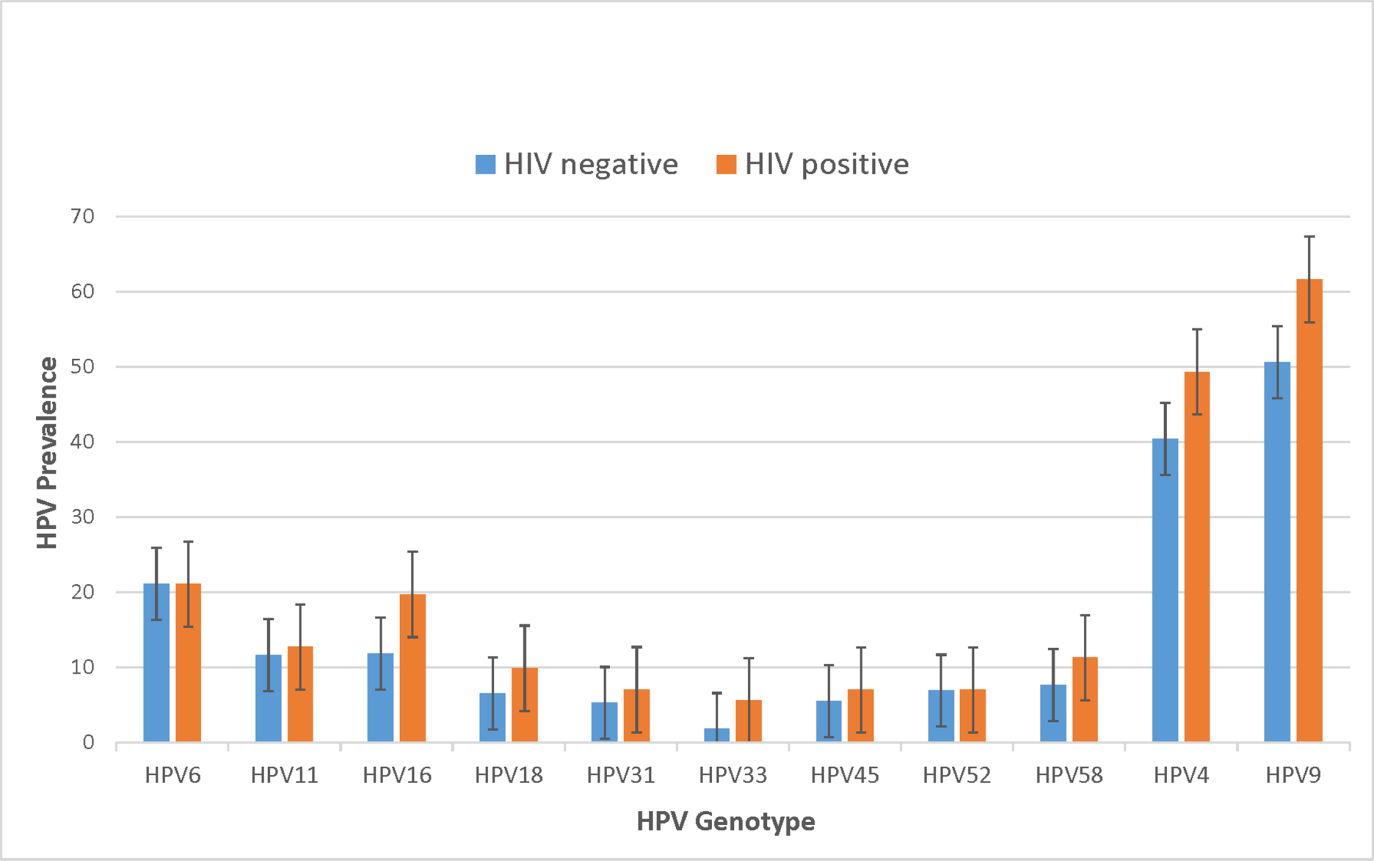
HPV prevalence by HIV status among Peruvian MSM and TW.

Of the 571 MSM and TW in the study who completed at least two study visits, 73 (12.8%) acquired HIV in 2 years, resulting in a 6% HIV incidence rate. Compared to those without HIV, participants with incident HIV were primarily younger than 25 (52% vs. 46%, *p* = .039) and more likely to have reported STI symptoms (44% vs. 29.4%, *p* = .038), more likely to have anal bleeding (28% vs. 11%, *p* = .005), and more likely to have anal ulcers (12% vs. 4%, *p* = .016) during the prior 6 months. Additionally, those with incident HIV were less likely to have used a condom during their most recent anal sex with a man (61% vs. 75%, *p* = .040), more likely to have ever had receptive anal intercourse (86% vs. 74%, *p* = .040), and more likely to have HRHPV (73% vs. 51%, *p* = .001). Differences in HPV prevalence (individual HPV types, HPV4, HPV9) by HIV status are shown in Fig 1.

We found no statistically significant differences between participants living with HIV and those without HIV in terms of sexual identity (*p* = .127), frequency of anal intercourse (*p* = .599), overall frequency of anal intercourse without a condom (*p* = .107), sex under the influence of alcohol (*p* = .654) or drugs (*p* = .876) in the prior month, engagement in transactional sex in the prior 6 months (*p* = .486), or circumcision status (*p* = .505).

In multivariate analyses, we adjusted for all statistically significant variables in Table 1. Model 1 shows the effect of HRHPV on HIV, Model 2 shows the effect of having HPV9 on HIV, and Model 3 shows the effect of HPV4 on HIV (Table 2). With the other variables remaining constant, the odds of HIV increased in the baseline presence of HRHPV genotypes by 181% (*p* = .004), HPV9 genotypes by 144% (*p* = .011), and HPV4 genotypes by 93% (*p* = .046). The odds were highest when the most HPV genotypes were present.

**Table 2:** Logistic regression of HIV status for 571 Peruvian MSM and TW in Peru.

	Model 1 (HRHPV)		Model 2 (HPV9)		Model 3 (HPV4)	
	*AOR*	95% CI	*AOR*	95% CI	*AOR*	95% CI
HRHPV	2.81**	1.38, 5.70				
Genotypes in HPV9 vaccine			2.44*	1.22, 4.87		
Genotypes in HPV4 vaccine					1.93*	1.01, 3.69
Condom used, last anal sex	0.40**	0.20, 0.78	0.40**	0.21, 0.78	0.41**	0.21, 0.79
Anal bleeding, last 6 months	3.05*	1.30, 7.13	2.84*	1.23, 6.57	2.77*	1.21, 6.34
Sexual role (passive vs. active)	2.67*	1.05, 6.77	2.71*	1.07, 6.87	2.48	0.98, 6.25
Tertiary education	0.84	0.43, 1.65	0.91	0.47, 1.77	0.91	0.47, 1.78
Age	0.97	0.91, 1.03	0.97	0.91, 1.03	0.97	0.91, 1.03
Pseudo *R*	10.2		9.4		8.4	

Circumcision did not play a role due to the small number of circumcised men with HIV. HRHPV, high-risk HPV; HPV9, 9-valent HPV vaccine; HPV4, 4-valent HPV vaccine.

*p < .05.

**p < .01.

In all three models, condom use during last anal sex with a man remained a protective factor against HIV infection, whereas anal bleeding was a risk factor. Engaging in receptive anal sex was a statistically significant risk factor for HIV in Models 1 and 2 and marginally significant in Model 3.

## Discussion

The overall annual HIV incidence rate of 6% in the study population is indicative of a troubling, continuing HIV epidemic in Lima, Peru, among MSM and TW. Our finding that participants who acquired HIV were more likely to have HRHPV confirms and extends previous research regarding the significant independent relationship between prevalent HPV (in general) and HIV acquisition among MSM [6]. Although previous work has linked the presence of unspecified single or two or more HPV genotypes, our work illustrates genotypes specific to incident HIV, including HRHPV, which can be part of standard testing, and the vaccine genotypes present in HPV9 and HPV4. Consistent with a large body of research, our data also linked receptive anal sex with risk of HIV acquisition [22].

Other studies have found significant relationships between genital warts, HPV, and multiple HPV infections and HIV-positive status [23,24]. Still, other research explained these relationships as a result of increased immunodeficiency and susceptibility to genital warts and HPV due to HIV infection [25–28].

This was a nonrandom sample of 600 MSM and TW who met eligibility criteria including genital wart status and were recruited at gay venues in metropolitan Lima, therefore, it is not representative of all MSM or TW in Lima. Some participants lost to follow-up at the final visit could have been HIV-positive, which would have affected our models of HPV and HIV infection. We also measured HPV status at a single point and combined anal and penile specimens into one combined sample. Retrospective studies can examine the relationship between HPV and HIV, although temporality may play a role (i.e., which infection, HIV or HPV, occurred first). Further, while this study found a significant relationship between having HRHPV and acquiring HIV, the presence of an association does not prove causality. As such, it is important to consider other explanations for this association. For instance, there may be other behavioral factors that may have predisposed participants to both HIV and HPV acquisition which we did not assess, such as sex toy sharing or douching. Further, there may have been biological reasons that partially explain why some participants were more prone to the acquisition of HR-HPV and HIV, including bacterial STIs. Lastly, it is possible that some participants were infected with HIV at baseline but tested negative due to the window period of the assay, and seroconversion may have driven higher HPV prevalence.

## Conclusions

Our findings support other research [6,29] that suggested vaccinating against HRHPV may have a protective effect against the acquisition of HIV among MSM and TW. Moreover, given the strong relationship between HRHPV and head, neck, throat, penile, and anal cancers, and data showing MSM are at increased risk of such cancers [25], HPV vaccination may benefit MSM and TW in this understudied population [30].

Although new vaccine adoption often takes more time in lower-resource settings, the HPV vaccine is currently provided exclusively to school-aged girls in Peru. This may help promote greater acceptance among vaccine decision makers and help expand coverage to school-aged boys and adult MSM and transgender individuals [31–33]. Comprehensive awareness-raising and health education efforts sponsored by the national government, health administrators, and civil society leaders regarding HPV and its consequences are important to keeping parents and communities informed, reassuring them the vaccine is safe and effective, and garnering community acceptance [31,34]. Early HPV vaccination paired with robust HIV prevention strategies can prevent both HPV related outcomes and incident HIV infection.
